# Key genes and drug delivery systems to improve the efficiency of chemotherapy

**DOI:** 10.20517/cdr.2020.64

**Published:** 2021-03-19

**Authors:** Zally Torres-Martinez, Yamixa Delgado, Yancy Ferrer-Acosta, Ivette J Suarez-Arroyo, Freisa M. Joaquín-Ovalle, Louis J. Delinois, Kai Griebenow

**Affiliations:** 1 Chemistry Department, University of Puerto Rico- Rio Piedras campus, San Juan, PR 00936, USA; 2 Biochemistry & Pharmacology Department, San Juan Bautista School of Medicine, Caguas, PR 00726, USA; 3 Neuroscience Department, Universidad Central del Caribe, Bayamon, PR 00956, USA; 4 Biochemistry Department, Universidad Central del Caribe, Bayamon, PR 00956, USA

**Keywords:** Cancer resistance, drug delivery systems, resistance genes

## Abstract

Cancer cells can develop resistance to anticancer drugs, thereby becoming tolerant to treatment through different mechanisms. The biological mechanisms leading to the generation of anticancer treatment resistance include alterations in transmembrane proteins, DNA damage and repair mechanisms, alterations in target molecules, and genetic responses, among others. The most common anti-cancer drugs reported to develop resistance to cancer cells include cisplatin, doxorubicin, paclitaxel, and fluorouracil. These anticancer drugs have different mechanisms of action, and specific cancer types can be affected by different genes. The development of drug resistance is a cellular response which uses differential gene expression, to enable adaptation and survival of the cell to diverse threatening environmental agents. In this review, we briefly look at the key regulatory genes, their expression, as well as the responses and regulation of cancer cells when exposed to anticancer drugs, along with the incorporation of alternative nanocarriers as treatments to overcome anticancer drug resistance.

## INTRODUCTION

Drug resistance and inefficient cancer therapy accounts for up to 90% of cancer-related deaths^[1]^. Resistance occurs when a cancer cell develops the ability to prevent chemotherapeutic drugs from entering inside the cell, or reduces the amount of the drug that can enter inside it to non-damaging levels. Current cancer management programs include surgery, radiation therapy, immunotherapy and/or chemotherapy (including toxic, non-targeted, and targeted therapy). The problem of drug resistance is highly sustained by intrinsic and extrinsic factors. Although many cancers initially respond successfully to chemotherapy, the development of drug resistance occurs in most patients^[2]^. The initial solution to the problem of resistance to single-agent chemotherapy is the combined administration of agents that have non-overlapping mechanisms of action.

Cancer cells have many growth mechanisms; they release proliferative signals while avoiding growth suppressor molecules to resist cell death. Anticancer agents cause DNA damage by targeting cellular replication as well as the growth signaling molecules of rapidly dividing cells^[3]^. Usually, cytotoxic drugs are cell-cycle specific and target a phase of the process. In this way, they normally induce mitochondria-mediated caspase-dependent apoptosis. For example, tamoxifen hormone targets the G1-phase, antimetabolites target the S-phase, podophyllotoxins target the G2-phase, and taxanes target the M-phase of the cell cycle^[4]^. In contrast, there are anticancer agents which modulate cellular process that are cell cycle-independent^[5]^. For example, alkylating and platinum-based agents can disrupt DNA at any stage of the cell cycle, and anthracyclines, as doxorubicin, interfere with DNA replication and mainly generates reactive oxygen species (ROS)^[5,6]^. However, anticancer drugs affect healthy cells and produce severe side effects. Depending on their mechanism, anticancer drugs can be divided into (1) alkylating agents, (2) antimetabolites, (3) mitotic spindle inhibitors, (4) topoisomerase inhibitors, (5) anthracyclines, and others^[7]^ [Table 1].

In chemotherapy, cytotoxic agents target different metabolic pathways, mainly that of the apoptotic machinery. Cancer cells can become resistant to cytotoxic agents and several cancer treatments due to the dysregulation of cell signaling pathways, as is described in several reviews[^1,7,23–28]^. One of the principal causes of drug resistance is the patient’s genetic predisposition, where the individual inherits genetic characteristics of an ineffective drug response. Another case of tumor drug resistance is the growth of cancer cells which adapt and survive after drug exposure. In this last scenario, exposure of tumor cells to the drug leads to resistance because of a genetic adaptation to the tumor microenvironment. Epigenetic modifications and an imbalance of random mutations influencing signaling processes also support carcinogenesis^[7,26,29]^.

Table 2 presents a list of the most well-known anticancer drugs used in chemotherapy for more than 20 years. Unfortunately, these anticancer drugs are not entirely effective and lead to the development of drug resistance to cancer treatment. Most genes presented in this review have developed resistance to one or more of these drugs.

Cancer-stem cells play an essential role in resistance to cancer treatment by promoting uncontrolled cell growth and generating tumors. Cancer-stem cells can self-renew and differentiate into multiple cell types. Such cells can persist in tumors as a distinct population, and can cause relapse and metastasis by giving rise to new tumors^[43,44]^. Furthermore, the tumor microenvironment can contribute to anticancer drug resistance, which decreases therapy effectiveness^[45,46]^.

Drug resistance can develop due to intrinsic genetic causes or can be acquired upon exposure to chemotherapeutic drugs^[47]^. Tumors with intrinsic resistance exhibit cell heterogeneity and inherent decreased responsiveness to chemotherapy^[1]^. On the other hand, acquired resistance is generated by most cancer patients under chemotherapy as a gradual decrease in drug efficiency^[47]^. Furthermore, in this type of resistance, mutations can affect the expression level of the drug target, affecting the structure of the protein (mostly receptors) and the target of the therapy Mutations can also affect other proteins within the cancer cells, which can become an oncogene, also known as a second proto-oncogenesis.

Cancer stem cells are also a result of mutations that turn them into a subset of cells within the tumor with a potential for self-renewal, differentiation, and tumorigenicity, making the tumor resistant to chemotherapy. Finally, chemotherapeutic drugs can also cause DNA damage in cancer cells and might increase the probability of the emergence of new mutations, including, for example, the activation of cell growth factors and cell defense systems^[1]^.

The multidrug resistance (MDR) syndrome impedes the efficiency of cancer treatments, and it can occur during or after the cancer treatment. MDR can result from a difference in the structure or mechanism of anticancer drugs. MDR’s principal causes include increases in the efflux activity of drug pumps and a decrease in drug transporters within the membrane^[48]^. MDR is common in cancers such as ovarian, breast, cervical, lung, prostate, and melanoma^[49]^. Development of MDR is the main cause for failure of the most widely used chemotherapeutic drugs (paclitaxel, cisplatin, docetaxel, vincristine, epirubicin, 5-fluorouracil, and oxaliplatin), and leads to cancer recurrence after one or more years of treatment^[50,51]^.

Some of the most well-studied cancer drug resistance mechanisms include drug inactivation, alteration of drug target, efflux pump, DNA damage repair, cell death inhibition, cancer cell heterogeneity, and epigenetics (explained in Table 3).

Researchers have suggested an alternative to reduce the possibility of developing acquired anticancer drug resistance. A patient’s biopsy sample of cancerous tissue can be screened to identify genetic anomalies that could lead to cancer treatment resistance^[55]^. This can contribute to determining the best suitable treatment, lowering the chances to acquire resistance after general chemotherapy sessions, and prevent the failure or risks of subsequent more toxic treatments. In addition to the standard pathological analysis, several clinicians have included these genetic screenings as part of the diagnostics to guide the selection of drug combinations on different types of cancers^[55]^. This approach could lead to a personalized therapeutic alternative based on the patient’s genetic pattern.

Investigations have been focusing on alternative drug delivery systems (DDS) designed to overcome cancer drug resistance. Efficiency in delivery and target specificity are the characteristics in consideration for drug delivery vehicle designs. DDS could increase bioavailability, diminish side effects, and improve therapeutic indexes when compared to current clinical drugs used for treatments^[27,56,57]^. Consequently, DDS could also help overcome acquired resistance induced by chemotherapy or radiotherapy^[58]^.

In this review, we summarize the most significant genes that contribute to drug resistance till date, discuss anticancer drug inefficacy, and present DDS as an alternative to overcome this clinical challenge.

## CANCER DRUG RESISTANCE RELATED GENES

Cancer cells can grow, develop, and survive in defiance of anticancer treatment due to intrinsic or acquired causes. Genes are key players to resistance to many common cytotoxic anticancer drugs. There is strong evidence pointing that most of these resistance-related genes are involved in DNA repair and apoptosis pathways^[59]^. In this regard, the most well-known and significant genes that contribute to anticancer drug resistance, based on our understanding, are outlined in this review. We present in the list of genes below their general information, the cancer types affected by drug resistance, how these genes are regulated in general, and recent research studies that incorporate drug delivery system techniques to combat cancer drug resistance.

### B-cell lymphoma-2 family proteins

Evasion of apoptosis supports the development of cancer, and it is an important resistance mechanism for cancer cells against chemotherapy. Apoptosis is characterized by two established pathways: an extrinsic pathway mediated by death receptors at the cell membrane and an intrinsic pathway mediated by the mitochondria. Gene products that influence apoptosis include B-cell lymphoma-2 (Bcl-2) family proteins. This large multigene family encodes proteins that are capable of inhibiting apoptosis (BCL-2, BCL-XL, BCL-W, BFL-1, BRAG-1, MCL-1, and A1) or promoting it (BAX, BAD, BAK, BCL-XS, BID, BIK, BIM, and HRK). In mammalian cells, Bcl-2, Bcl-xL, Bcl-w, Mcl-1, and A1, are the Bcl-2 proteins that block the apoptosis promoting proteins Bak and Bax, inhibiting their action by interacting with them^[60–62]^. The cellular outcome of undergoing intrinsic apoptosis or surviving depends on the balance and interaction between the pro- and anti-apoptotic proteins inside the cell. These Bcl-2 proteins show four homologous domains in their sequence (BH1, BH2, BH3, and BH4) and are called BCL-2 homology motifs^[63,64]^, except the BH3-only proteins; Bim, Bid, and Bad. Genetic alterations associated with cancer and tumor growth often affect programmed cell death regulation in a way that favors cell proliferation^[65]^. These genetic changes are either inherited or acquired during the cell cycle. These include substitutions, insertions, or deletions of small or large fragments of DNA, genomic amplification, and rearrangements^[66,67]^. For example, chromosomal translocation t (14;18) activates the BCL-2 gene in most non-Hodgkin’s lymphomas^[68,69]^; nucleotide substitution and a frameshift mutation^[70]^ inactivates the BAX gene in some colon, hematological, and stomach malignancies^[71–73]^; retrovirus gene insertion activates BCL-XL gene in murine leukemia^[74]^. These BCL-2 family gene alterations result in overexpression of either apoptosis-suppressing or apoptosis-inducing proteins of the Bcl-2 family. Similarly, the Bcl-2 protein is overexpressed in numerous breast and prostate cancers^[75–77]^. Other studies have shown that mutations found on the coding sequence of the BCL-2 gene in patients at the time of diagnosis were associated with a shortened time to its transformation into an aggressive lymphoma, and subsequently, earlier death due to the lymphoma^[78]^.

Additionally, it was found that the Bcl-2 protein induces cell migration and invasion in a breast cancer cell line and also promotes metastasis to the lungs in a mouse model^[79]^. BCL-2, MCL-1, and BCL-XL are also overexpressed in several non-small cell lung carcinomas (NSCLC)^[80–82]^. Amplification of the apoptotic inhibitors Bcl-2-like genes, MCL-1 and BCL-XL, and deletion of apoptotic-promoter genes BOK and PUMA are presented in the somatic copy number variations in over 3000 cancer specimens across 26 human cancer types^[83]^.

Besides malignancy, the imbalance ratio between apoptosis-suppressing and apoptosis-inducing proteins of the Bcl-2 family often makes cancer cells more resistant to a number of cell death inducers, including chemotherapeutic drugs, by impeding drug-induced damage from successfully translating into cell death^[84]^. Multidrug resistance (MDR) is reported to be associated with the overexpression of specific proteins such as P-glycoprotein and the anti-apoptotic genes of the BCL-2 family, where the former plays the role of expelling the drug out of the cells while the latter induces their proliferation^[85]^. Bcl-2 affects cancer drug resistance by inhibiting the apoptotic effect on cancer cells by dimerizing Bax and Bad, pro-apoptotic members of the Bcl-2 family. Also, overexpression of Bcl-2 can prevent chemotherapy treatment by blocking paclitaxel-induced apoptosis, and the translocation of nuclear factor of activated T lymphocytes. Additionally, BCL-2 antagonizes apoptosis induced by drugs through the inhibition of calcineurin protein activation, thereby preventing the activation of T cells from the immune system^[86]^.

In breast cancer cells, overexpression of Bcl-2 has been correlated to the formation of polyploid cells, which confer MDR properties to cancer cells^[51]^. In colorectal cancer, it has been shown that the cytokine interleukin 17 (IL-17) plays an important role in promoting the development of resistance to cisplatin by inhibiting the expression of several pro-apoptotic proteins, including those from the Bcl-2 family such as Bax^[87]^. In breast cancer, IL-17 has also been shown to promote paclitaxel resistance through activation of the ERK1/2 pathway^[88]^. Defects in splicing events lead to resistance against selected therapy agents. Studies provide evidence that BIM alternative splicing products play a key role in drug resistance. In one study, inhibition of the three major protein products (BimEL, BimS, and BimL) resulted in different levels of resistance to glucocorticoid treatment in acute lymphoblastic leukemia cells^[74]^. Using paired-end DNA sequencing, Ng *et al.*^[89]^ (2012) discovered an intronic deletion polymorphism in BIM that was sufficient to confer intrinsic resistance to the tyrosine kinase inhibitors, imatinib and gefitinib in chronic myeloid leukemia (CML) and epidermal growth factor receptor-mutated non-small-cell lung cancer (EGFR NSCLC). In summary, these studies point at targeting the anti-apoptotic members of the Bcl-2 family as a strategy to prevent MDR.

### Chromodomain-helicase-DNA-binding protein 4

Chromodomain-helicase-DNA-binding protein 4 (CHD4) is the main component of the nucleosome-remodeling and histone-deacetylation (NuRD) complex. The NuRD complex’s primary function is to regulate gene expression and promote DNA repair. This complex is expressed throughout all tissues and is composed of multiple subunits, including ATP-dependent chromatin remodeling helicases CHD3/CHD4. The NuRD complex contributes to several cellular processes such as stem cell differentiation, cell cycle regulation, genome integrity maintenance, DNA damage repair, and development of the immune system^[90]^. NuRD subunits contribute to oncogenesis and cancer progression through DNA-damage repair, impacting the tumor’s microenvironment^[91]^.

The CHD4 gene plays a critical role in epigenetic transcriptional repression^[91]^. This gene has been associated with oncogenic effects such as promoting cancer cell sternness, renewal and altering cell-cycle^[92]^, and poor prognosis of advanced-stage cancer^[91]^. In collaboration with the histone deacetylases (HDACs), which allow the histones to wrap the DNA more tightly, and DNA methyltransferases, which mostly repress genes, CHD4 contributes to silencing as well as reducing and blocking the transcription of tumor suppressor genes. One of the main reasons for tumor recurrence is the resistance to DNA damage, and genes such as CHD4 enable this repair in cancer cells. CHD4 promotes DNA repair from insults such as oxidative damage in cancer cells^[93,94]^.

Drug resistance is promoted in cancers associated with breast cancer genes (BRCA), which are sensitive to DNA-damaging agents, once the CHD4 expression decreases. Furthermore, CHD4 expression reduction affects cancer cell’s autophagy process as well as the ERBB2 gene, which is an epithermal growth factor member, resulting in a drug resistance effect^[93]^. Expression of CHD4 can increase stem-cell characteristics in cancer cells, stimulating anticancer drug resistance to DNA-damaging drugs^[44]^. CHD4 can regulate cancer cell behavior through post-transcriptional modifications. CHD4 is associated with transcriptional repression of genes involved in the repair of double-strand break DNA-damage. It has been considered a potential biomarker present in biopsies of patients (with significant upregulation) in cancers such as liver, renal, osteosarcoma, breast, and ovarian^[93]^.

Wang *et al.*^[93]^ (2019) showed that CHD4-increased expression was associated with advanced tumor invasion during metastasis and increased vascularity, promoting a more aggressive cancer phenotype. This group also reported that increased expression of the CHD4 gene was proportional to cancer treatment resistance by suppressing the expression of the cell cycle inhibitor and anti-proliferative effector, p21, which works together with the DNA-repair gene BRCA to cause an overall decrease in the sensitivity of cells to anticancer treatment. Furthermore, a decrease in the gene expression of CHD4 promotes radiotherapy sensitivity of head and neck cell carcinoma. CHD4 cooperates with DNA methyltransferases (DNMTs) in the silencing of many tumor suppressor genes; therefore, its decreased expression inhibits cell proliferation and sensitizes cells to radiotherapy^[93,94]^. In ERBB2^+^ breast cancer cells, which are resistant to Trastuzumab, a monoclonal antibody anticancer treatment, the depletion of CHD4 was shown to induce the cell’s sensitivity to this antibody by reducing ERBB2 signaling, affecting the autophagy process, and decreasing cell proliferation^[93,95]^.

The CHD4 gene has a crucial role in colorectal cancer, and it is important to consider the activity of this gene to establish a treatment for colorectal cancer patients^[93]^. Overexpression of CHD4 led to pronounced radiotherapy-resistance by maintaining DNA hypermethylation transcription silencing on colorectal cancer patients^[96]^. In addition, CHD4 knockdown increased the chemosensitivity of breast cancer cells towards cisplatin^[94]^ and increased the sensitivity of hepatocellular carcinoma cells towards epirubicin, an antitumor antibiotic^[44]^.

The DNA-repair promoting gene, CHD4, is responsible for the transcriptional activity of the anti-proliferative gene, cyclin-dependent kinase inhibitor 1 (CDKN1A or p21); therefore, these genes have opposed functions regarding cell survival. CHD4 deficiency debilitates cell survival by not-suppressing and increasing p21 levels^[94]^. Inhibition of CHD4 results in the restoration of p21 expression and recovery of breast cancer cell sensitivity to cisplatin and poly ADP ribose polymerase (PARP) inhibitors^[93]^.

Unfortunately, knockdown of CHD4 subunits can negatively affect the chromatin-remodeling ability of the NuRD complex, promoting cell proliferation, migration, and invasion, which represses apoptosis pathways and allows cancer cells to resist drugs that lead to DNA-damage^[91]^. Therefore, if CHD4 inhibitors are therapeutically tested, a targeted drug delivery system must be developed to direct this drug into the tumor to decrease the chances of affecting healthy cells or other unwanted secondary effects. Many efforts have been made in the development of therapeutic strategies against cancer that are likely to develop resistance. The combination of radiotherapy, together with an inhibitor of the NuRD complex subunit CHD4, should be a viable alternative to treat colorectal and liver cancer^[44,91,93]^.

### p53

TP53 was the first tumor suppressor gene identified in 1979. Since then, this gene has been extensively studied. p53 works mainly as a transcription factor, and its most important function is to induce or suppress the transcription of effector genes that will inhibit cancer cell proliferation, promote apoptosis, and impede tumor development^[97]^. DNA integrity is maintained by p53 through activation of the transcription of genes inducing cell cycle arrest as a DNA damage response^[40]^. Once DNA damage is detected in the cell, p53 favors the elimination of the affected cell, inducing activity of pro-apoptotic genes such as FAS (Fas Cell Surface Death Receptor) and BAX (from the BCL-2 family), and downregulating anti-apoptotic genes such as BCL-2^[40]^. The activation of p53 occurs in response to cellular stress and can induce cell cycle arrest to ensure genomic integrity^[97–99]^. Once p53 is activated, several effectors and p53-responsive genes such as CDKN1A, GADD45α, p21, MDM2, and RIT42, among others, work to inactivate cyclin-dependent kinases on the cell cycle^[99]^. Cancer drug resistance is influenced by loss-of-function p53 gene mutations, affecting mainly its transcriptional activity. The function of p53 is lost through the modifications that the gene undergoes (i.e., single point mutations and some hotspot mutations), leading to sensitivity loss to cytotoxic gents^[100]^

In contrast, we also found studies that show gain-of-function p53 mutations by inducing new interactions with other transcription factors that further promote chemoresistance^[101]^. Mutations of the p53 gene will obstruct cancer treatment. Hypoxia promotes upregulation of p53 in cancer cells blocking the cell cycle, and this event leads to the downstream activation of the p21 gene, decreasing the cytotoxic effect of anticancer drugs like cisplatin^[102]^. An in-vitro study reported that when p53 is mutated on cancer cells, anticancer drug 5-fluorouracil sensitivity is reduced^[103]^. The most common cancer types affected by the mutation of p53 are ovarian serous carcinoma, lung cancer, pancreatic cancer, head and neck squamous cell carcinoma, and breast carcinoma^[104]^.

Studies have found small molecules that can restore the conformation and function of mutated p53 to prevent drug resistance. These include derivatives of the thiosemicarbazone family, PRIMA-1 and MIRA-1^[100]^. Also, treatment with blockers of p53-inhibitory proteins such as MDM2 could help restore p53’s function in the cases where there is an under-expression of p53 or an overexpression of MDM2^[100]^. To recover p53’s decreased function that leads to drug resistance, interventions with nanomedicine to deliver small molecules or MDM2 inhibitors, plus the specific treatment against the tumor, could help advance the battle against cancer drug resistance.

### Cyclin-dependent kinase inhibitor 1

**Figure 1.**
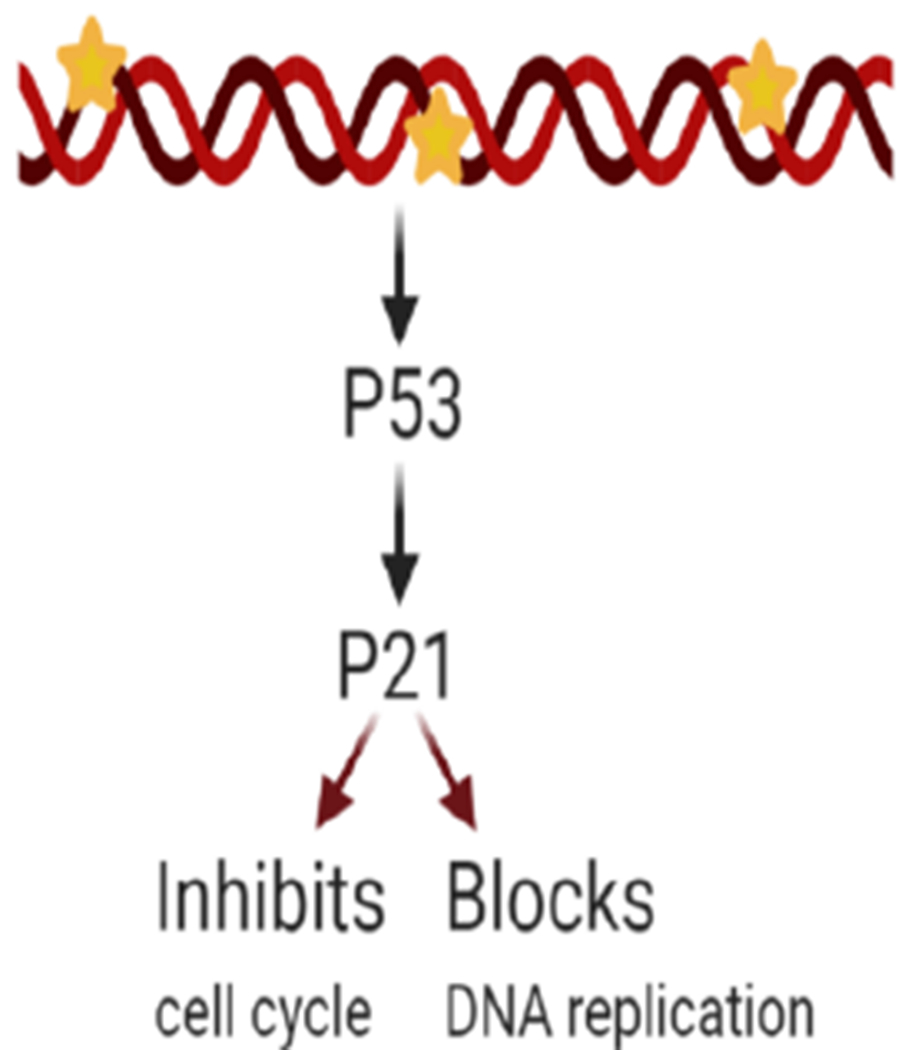
p21 overexpression effects after DNA damage. Excess p21 induces cell cycle inhibition or blockade of DNA replication

Cyclin-dependent kinase interacting protein 1, also known as p21, is encoded by the CDKN1A gene. p21 is capable of controlling cyclin complexes, including Cycling dependent kinase 2 (CDK2), a catalytic subunit that can restrict cell cycle and DNA replication. In healthy cells, p21 prevents proliferation, while in several cancer cells, this function is dysregulated. Among p21 functions, its role in maintaining genomic stability, DNA-damage repair, apoptosis, and tumor-suppressing functions is worth mentioning^[105]^. In cancer cells, p21 functions as a tumor suppressor and an anti-apoptotic protein, and its relationship with the tumor suppressor protein p53 have been under study due to its potential contribution to cancer therapy. Studies report various roles for p21, depending on its subcellular localization. P21 can be considered as an oncogenic protein inside the cytoplasm, while it can operate as a tumor suppressor inside the nucleus^[106]^. As part of an anti-apoptotic protein, p21 can promote cancer tumor evolution and growth by diminishing DNA damage accumulation^[107]^. A study incorporated human leukemia cells treated with SP600125, an anti-inflammatory and anticancer drug that inhibits c-Jun N-terminal kinase, to generate an increase in p21 expression as well as p21 phosphorylation, thereby preventing its binding with proliferating cell nuclear antigen, a DNA polymerase cofactor, while also inactivating caspase-3 and consequently apoptosis^[95]^. The anti-apoptotic role of p21 is inhibiting the ability of pro-apoptotic proteins to affect apoptosis. Differentially, p21 in the nucleus has a tumor suppressor role due to the regulations to the cell cycle on CDK/cyclin complexes suppression. In a study, p21 and p53 were introduced through a nanoparticle injection, and cells were introduced into a breast cancer mouse model resulting in a reduction in cell proliferation and tumor growth^[108]^. Once the DNA is damaged, an increase in p53 levels leads to the activation of p21 transcription. Subsequently, p21 can either inhibit cell cycle binding CDK/cyclin complexes or block DNA replication via its interaction with DNA polymerase cofactors^[109]^ [Figure 1].

The p21 and p53 relationship has been under investigation to consider treatment for cancer cell drug resistance. p21 mediated p53-dependent apoptotic pathways, and p53-independent pathways^[110,111]^ have been recently studied. These pathways lead to transcription induction of p21 and DNA-damage in cancer cells^[112]^. Reduced levels of p21 are associated with tumorigenesis on several cancers such as squamous cell carcinoma of the lung, colorectal, ovarian, cervical, and head and neck^[113]^. P21 is an important downstream target of p53 and is rarely mutated. This means that the resistance induced by p21 could be a consequence of different factors: deficiency of the p21 gene^[114]^, or high expression of cytoplasmic p21; and as a result, the p21 binding to procaspase-3 blocking caspase cascade and apoptosis^[115,116]^. However, p21 overexpression is also correlated to the aggressiveness and invasiveness of different cancers [Figure 1]^[109]^. Other studies have reported that p21 collaborates with anticancer, DNA-damaging agents to promote cell cytotoxicity. DNA-damaging agents can be combined with the anti-apoptotic p21 function as a possible target for anticancer treatment^[105]^. Considering the controversy around p21’s various responses, more research is needed to further understand its mechanism of action on specific cancer types. Research is being conducted to systemically study the regulation of p21’s expression upstream and downstream at different levels (transcriptionally, post-transcriptionally, and post-translationally) and contribute with therapeutic approaches against cancer and drug resistance treatments^[117]^.

### Multidrug resistance gene or P-glycoprotein-1

The multidrug resistance gene or P-glycoprotein-1 (MDR1) gene is responsible for the expression of P-glycoprotein (P-gp), a transmembrane glycoprotein that mediates ATP-dependent efflux with permeability properties to expulse cytotoxic drugs into the extracellular space^[118]^. Multidrug resistance protein 1 is a member of the ATP-Binding Cassette (ABC) transporter protein family. ABC transporters are transmembrane proteins that move compounds into or out of the cell. These transporter proteins are composed of a pair of transmembrane domains and two nucleotide-binding domains. They are essential in the elimination of toxins from the human body. MDR1 is normally expressed in healthy tissue (usually on the liver, kidney, colon, pancreas, uterus, placenta, testis, and brain), although its overexpression has been associated with cancerous cells^[119,120]^.

Before treatment, it is important to evaluate the presence of MDR1 mutations in cancer patients to predict the tumor’s sensitivity to therapy. Patients with mutated MDR1 can be intrinsically resistant to drugs or could develop resistance over time^[121]^. It is worth mentioning that when P-gp is pharmacologically inhibited, thyroid hormones can promote its transcription and its function in the body^[122]^. MDR1/P-gp1 causes resistance to chemotherapeutic agents in different ways; for example, by direct interaction, in the case of paclitaxel and doxorubicin, and by indirect interaction, in the case of cisplatin, carboplatin, and oxaliplatin^[123]^. The overexpression of MDR1 contributes to drug resistance, particularly when genetic polymorphism variations are present. MDR1 G1199A variation exhibits a serine-to-asparagine transition in amino acid 400 in a Pgp cytoplasmic domain, producing an alteration on the efflux and transepithelial transport as well as drug sensitivity to chemotherapeutic agents^[124]^.

Consequently, overexpression of P-gp decreases intracellular anticancer drug accumulation, which helps prevent the generation of MDR^[121]^. An increase in the expression of MDR1 by vitamin C has also been associated with the inhibition of the anti-tumor action of doxorubicin in ovarian and prostate cancer cells^[17]^. Recently, a study defined the resistance mechanism of paclitaxel and olaparib (inhibitor of PARP1) in resistant ovarian cancer cells that was reversible with the MDR1 inhibitors, verapamil and elacridar. They found that paclitaxel-resistant cells were cross-resistant to Olaparib, Rucaparib (PARP inhibitors), and doxorubicin, but not to the PARP inhibitors, Veliparib or AZD2461^[123]^.

MDR1 gene expression can also be regulated through small interfering RNA (siRNA), which are lower in toxicity to healthy cells, and show higher specificity to the cells containing the mutated gene. This targeted siRNA therapy downregulates the MDR1 gene transcription, leading to a decreased amount of P-gp transporter proteins and a reduction of anticancer drug expelled from the cell^[119]^. Other strategies to decrease the efflux of anticancer drugs through P-gp consist of developing compounds that either compete with anticancer drugs for transport or act as direct inhibitors of P-gp. Up to date, no P-gp blockers are being used in the clinic, possibly due to the toxic effects of such inhibition. Several alternative approaches could include nanotechnology to specifically target the cancer cells and deliver P-gp inhibitors, molecules that reduce the expression of P-gp in cancer cells, or anticancer drugs.

### Glioma pathogenesis-related protein 1

The glioma pathogenesis-related protein 1 (GLIPR1) is a member of the cysteine-rich secretory proteins (CRISPS), consisting of the following members: antigen 5 (Ag5), and pathogenesis-related 1 protein (Pr-1) CAP superfamily containing three core members, GLIPR1, GLIPR1-like 1 (GLIPR1L1), and GLIPR1-like 2 (GLIPR1L2)^[125]^. GLIPR1, a p53 target gene cluster found on human chromosome 12q21, is located in the endoplasmic reticulum (ER) membrane, and it is involved in the ER secretory pathway^[126,127]^. GLIPR1 is reported to contain an amino-terminal peptide sequence and a transmembrane domain that indicates its secretion or its location on the surface of the cell membrane^[128]^.

Downregulation of GLIPR1 in prostate cancer and other malignant cell lines has been observed, largely in part to the methylation of the human GLIPR1 promoter^[129]^. Initially identified as a tumor-suppressor gene with apoptosis-inducing activities in prostate cancer, GLIPR1 pleiotropic effects have been reported to be highly expressed and upregulated, and it acts as an oncogene specifically in glioblastomas and gliomas, thus promoting cell proliferation^[127,130,131]^. The underlying mechanism of upregulated GLIPR1 cell growth stimulation has been studied in human lung adenocarcinoma A549 cells and correlates with inducing anti-apoptotic Bcl-2 protein expression^[130]^. In glioma cells, GLIPR1 overexpression reduced c-Jun N-terminal kinase (JNK) phosphorylation and induced Bcl-2 expression, thus increasing cell survival and glioma cells’ protective effect against apoptotic stimuli such as Fas ligation, chemotherapy, and radiation treatment^[132]^. Conversely, the mechanism contributing to GLIPR1-induced apoptosis is dependent on Bcl-2 downregulation and phosphorylation at Thr56 and Ser70, which support p53-induced apoptosis; and on the increase in ROS, signaling by apoptosis signal-regulated kinase 1 (ASK1), mitogen-activated protein-extracellular signal-regulated kinase kinase (MEK), and the consequent activation of JNK. Thereby, GLIPR1 acts through the ROS-ASK1-MEK4/7-JNK signaling pathway^[133,134]^. Moreover, GLIPR1-mediated apoptosis through the Bcl-2 family proteins and caspases may occur through caspase-dependent and caspase-independent pathways^[134]^.

Originally identified as a tumor-suppressor gene with apoptosis-inducing activities in prostate cancer, GLIPR1 has been reported to be upregulated in glioblastomas, enhancing cell proliferation^[127]^. The mechanism contributing to GLIPR1-induced apoptosis is associated with an increase in ROS and consequent activation of the c-Jun N-terminal kinase (JNK) pathway^[133]^.

Downregulation of c-Myc protein and CK1a-mediated targeted destruction of c-Myc and b-catenin in prostate cancer cell lines contributes to apoptosis induction by GLIPR1. Also, serine/threonine-protein kinase AURKA and Xenopus kinase-like TPX2 protein signaling pathway suppression by GLIPR1 interaction with heat shock cognate protein 70 (Hsc70) also contribute to apoptosis induction^[135]^. TPX2 has been associated with metastasis and the prognosis of bladder cancer. New findings have identified GLIPR1 as part of a regulatory circuit composed of TPX2 and p53, which modulates cell proliferation, migration, invasion, and tumorigenicity of bladder cancer cells.

The GLIPR1 gene has been identified in different forms of human cancers, including prostate, lung, ovarian, Wilms’ tumor, acute myeloid leukemia, and in the most aggressive types, brain cancer, glioblastoma multiforme/astrocytoma, and within glioma cell lines^[133,136]^. Overexpression of GLIPR1 induces apoptosis in prostate and lung cancer cells. In contrast, GLIPR1 overexpression in glioma and osteosarcoma cells leads to an increase in the proliferation, survival, invasion, migration, and anchorage-independent growth^[130,131,137]^. In a study by Dong *et al.*^[137]^ (2015), overexpression of GLIPR1 induced the differentiation of osteosarcoma cancer-initiating cells and upregulated miR-16, thus blocking anti-apoptotic BCL-2 genes. GLIPR1 promotes an increase in Bcl-2 expression to subsequently decrease the apoptosis of A549/DDP lung cancer cells. The upregulation of GLIPR1 increases and affects drug resistance by promoting cell proliferation. Otherwise, if GLIPR1 is silenced in A549/DDP cells, caspase-3 dependent apoptosis is induced by mitochondrial signaling pathways through the decreased expression of the Bcl-2 protein^[130]^.

Downregulation of GLIPR1 and gene knockdown experiments in various leukemia cell lines treated with the small drug SB225002 (N-(2-hydroxy-4-nitrophenyl)-N’-(2-bromophenyl)urea) resulted in elevated production of ROS, a decrease in cell proliferation linked to an increased level of apoptosis due to GLIPR1 silencing, and amplified drug resistance^[138]^. In another study, the siRNA-mediated knockdown of GLIPR1 expression induced a reduction in the number of melanomas, glioma cell invasion and proliferation^[136]^. In human lung adenocarcinoma A549 cells, upregulation of GLIPR1 stimulated cell proliferation by inducing the increased expression of Bcl-2, thus increasing resistance to the chemotherapeutic drug cisplatin^[130]^. To increase the apoptotic effects of docetaxel in prostate cancer cells and overcome resistance, synergistic treatment with recombinant GLIPR1 (GLIPR1-DTM) inhibited tumor growth, consequently enhancing the chemotherapy effect^[139]^. In summary, a decrease in GLIPR1 expression is another recommended strategy to diminish resistance to anticancer drugs such as cisplatin and docetaxel.

### Human epidermal growth factor receptor-2

The ERBB family comprises four receptor tyrosine kinase members named EGFR, ERBB2 (HER2), ERBB3 (HER3), and ERBB4 (HER4), located on the cell surface. These four members share structure similarities, such as an extracellular binding domain, a transmembrane lipophilic segment, and an intracellular tyrosine kinase domain^[140,141]^. Of the ERBB family, HER2 is a proto-oncogene located on the long arm of chromosome 17, whose activation relies upon homodimerization when expressed at high levels and by hetero-dimerization with EGFR or kinase-inactive HER3]^[141–145]^. After ligand binding, intracellular cell signaling pathways result in the inhibition of apoptosis, promoting proliferation, and tumorigenesis^[146]^. The molecular mechanisms of HER2-mediated tumorigenesis encompass various models, including the overexpression of HER2, which induces an increase in the HER2-containing dimers, maximizing, and sustaining signaling activity^[147]^. Among the dimer complexes formed, HER2/HER3 is the most critical activator of the PI3K/Akt signaling pathway (crucial for cell survival)^[148]^. Transcript variants of HER2 manifest higher dimerization, increased ligand-independent signaling activity, and a significant presence in HER2 amplified tumors^[147,149]^. Activation of src kinases, second messengers of HER2, exhibit increased Src protein levels and protein kinase activity in many human tumor tissues when combined with EGFR, to yield a synergistic tumorigenic effect^[147]^. HER2 involvement in G1/S cell cycle checkpoint control is regulated by cyclin D1 and its cyclin-dependent kinases (CDK), which play a critical proliferative role in cell cycle progression, and the CDK inhibitor p27 as a cell-cycle regulator through the induction of G1 arrest, halting cell growth^[150,151]^. HER2 tumorigenic signaling also appears to be potentiated by a stable interaction via one of two EGF-like domains with the transmembrane mucin glycoprotein Muc4, known to frequently display an altered expression in many cancer types, thus promoting tumor cell proliferation and metastasis^[152,153]^. Gene amplification of HER2 is known to occur in a variety of tumor types and in approximately 25% of human breast cancers, where it manifests as an early event^[141,144]^. HER2 gene amplification is the primary mechanism prior to protein overexpression of HER2, consequently activating the HER2 signaling network leading to uncontrolled cell proliferation and poor prognosis^[154–156]^. HER2 overexpression and activity drive a tumorigenic signaling cascade in breast cancer when homodimerization and HER2/HER3 heterodimerization events arise^[157]^.

HER2 overexpression has been associated with resistance to chemotherapeutic agents^[158]^. This has been observed in malignancies other than breast cancer such as gastric, ovarian, colon, lung, cervical, pancreatic, and esophageal cancers; presenting, in general, a more aggressive disease, a lower survival rate, and a higher recurrence risk^[141,159]^. In the case of HER2 knockdown, reduced proliferation and apoptosis induction was observed *in vitro* on breast cancer tumors that overexpressed HER2; also, tumor regression after HER2 silencing with shRNA has been observed using *in vivo* a mouse xenograft model^[147,160]^. The potential of HER2 as a target for cancer therapeutic strategies mostly involves the use of various antibody-based agents and tyrosine kinase inhibitors (TKIs), either as single agents or in combination with other therapies.

The upregulation of HER2 in metastatic breast cancer to the uterus, in combination with tamoxifen therapy, stimulates aggressive growth and invasiveness of tumors, as HER2 overexpression is associated with relative resistance to tamoxifen, and increased sensitivity to anthracycline chemotherapy, usually 5-fluorouracil and doxorubicin^[161–164]^. In another study, treatment with gemcitabine (GEM) enhanced HER2 expression on low HER2 expression breast cancer cell lines, while paclitaxel treatment induced a low and moderate HER2 upregulation. Related studies in HER2-positive breast cancer cells demonstrated that overexpression of HER2 induced paclitaxel chemotherapy resistance^[165]^. The therapeutic outcome of the monoclonal anti-HER2 antibody-drug, Trastuzumab, is known to downregulate HER2 signaling PI3K/Akt and MAPK pathways and exhibit primary resistance in HER2-positive tumors as a monotherapy^[166]^. Some breast cancers even contain an abnormal form of HER2, lacking the extracellular domain needed for Trastuzumab binding, thereby causing resistance to the drug^[141]^. To overcome resistance, conjugation of Trastuzumab with the cytotoxic agent emtansine (T-DM1) requires elevated HER2 expression levels. Thus, pretreatment with GEM was used to increase HER2 upregulation, and T-DM1 binding to HER2 on breast cancer cell surface was used as a strategy to induce antiproliferative effects^[167–170]^.

In malignant pleural mesothelioma (MPM) cancer cells, the TKIs, lapatinib and afatinib, prevented cell proliferation, upregulating and downregulating HER2 expression, respectively. Furthermore, lapatinib enhanced the monoclonal anti-EGFR antibody drug cetuximab and Trastuzumab binding with MPM cancer cells. As a result of heightened cetuximab- and Trastuzumab treatment, antibody cellular cytotoxicity (ADCC) in MPM cell lines was observed. Likewise, lapatinib enhances Trastuzumab-mediated ADCC in HER2-positive breast cancer and esophageal and gastric cancer cell lines^[148,168]^. Cisplatin is the standard treatment for gastric cancer; however, high expression of HER2 is associated with resistance to cisplatin-based chemotherapy^[171]^. An improvement to HER2 downregulation, as well as an increased tumor cell binding and blockade of ligand-dependent and independent- tumor growth, was accomplished with the use of the antibody ZW25^[172]^. In summary, several strategies can be used to target HER2’S cancer drug resistance effects, from antibodies such as ZW25 to disulfide bond disrupting agents such as RBF3 or a combination of drugs that allow HER2-overexpressing cells to regain their sensitivity to tamoxifen or cisplatin.

### N-myc downstream-regulated gene

Cancer metastasis is the process in which cancer cells from an organ disseminate to another through circulation^[173]^. The N-myc downstream-regulated gene (NDRG) family has been identified as one of several metastasis suppressors involved in cancer cell invasion. The NDRG family of proteins contains four members: NDRG 1-4. The family functions are not well known, but they are associated with tumor suppression, cell proliferation, and stress response^[174]^. NDRG1 has shown to be an iron-regulated growth suppressor and metastasis inhibitor, exhibiting anti-oncogenic activity, decreased cell proliferation, migration, invasion, and angiogenesis^[175]^. NDRG1 is mainly located in the cytoplasm and translocates to the nucleus after DNA damage, hypoxia, and cell differentiation signals^[176]^. This protein is a downstream target of p53, and it is involved in cancer cell resistance to hypoxia and retinoic acid (anticancer activity and chemo-preventive properties)^[177]^. Nevertheless, it has not been established if NDRG1’s expression is inversely related to the survival of cancer cells^[178]^. In one study, NDRG1 demonstrated its capacity to suppress metastasis progress without altering tumor progression in an *in vivo* prostate cancer model^[179]^. NDRG1 has a pleiotropic behavior, considerable similar effects were observed on colon and pancreatic cancer^[180,181]^.

NDRG1 can associate with other genes and proteins such as KAI1 and ATF3. NDRG1 expression is elevated in non-small cell lung carcinoma and contributes to cancer growth while having a variety of functions. NDRG1’s overexpression reduces anticancer drug-induced cytotoxicity in lung cancer by downregulating the stress-inducible gene ATF3. The ATF3 protein, located in the cytoplasm and nucleus, promotes apoptosis and inhibits cisplatin-induced cytotoxicity in lung cancer A549 cells^[182]^. Thus, by inhibiting ATF3’s cisplatin-induced cytotoxicity, NDRG1 can also regulate anticancer drug sensitivity to cisplatin. On the other hand, suppression of NDRG1-mediated metastasis occurs upon loss of KAI1 expression *in vitro* and *in vivo,* demonstrating that KAI1 is a functional downstream target of the NDRG1 pathway on prostate cancer^[179]^. These results suggest that inhibition or suppression of KAI1 could also be a target to decrease NDRG1’s mediated anticancer drug resistance.

### Hypoxia-inducible factors

Hypoxia induces chemoresistance by two major factors: (1) low drug concentration in hypoxic cells and (2) impaired cell proliferation of hypoxic cells by starvation^[183]^. When hypoxia is induced through carcinogenic pathways, the cellular response is mediated by hypoxia-inducible factors (HIF-1α, −2α −3α, and −β). Hypoxia-inducible factors (HIFs) are transcription factors that form heterodimers. The α -subunit implies degradation and sensitivity to oxygen, the β-subunit means oxygen independence and the −3α serves as a suppressor or negative regulator for HIF-1α and HIF-2α (tumor promoters due to cellular response to low oxygen). HIFsare involved in different cancer stages - HIF-2α is responsible for chronic and prolonged phases of metastasis and anticancer drug resistance that occur in later stages of cancer; whereas HIF-1α is involved in the early stages of cancer that later can switch to HIF-2α through the upregulation of signaling proteins. HIF-1α and HIF-2α function can overlap during tumor development^[184,185]^.

HIF-2α is a transcription factor localized in the cell nucleus, and it is expressed under hypoxic stimulation. HIF-2α activation controls the intracellular hypoxic response around the body due to its expression in endothelial, parenchyma, and interstitial cells in multiple organs. HIF-2α can modulate the expression of cytochrome c oxidase isoforms to enhance the electron transport chain. Because HIF-2α is expressed in multiple organs, it affects many different types of cancer. The cancer types affected as a consequence of low oxygen availability in cellular and organismal levels are breast, colon, ovarian, pancreatic, prostate, renal, and hepatocellular cancers. The solid tumor cancer types, where HIF-2α is frequently detected, include: head and neck, renal, bladder, glial, breast, ovarian, prostate, and the digestive system^[186]^.

HIF-2α’s most important role is to control vascular morphogenesis, integrity, and assembly; and mediate p53’s suppression to maintain the human embryonic stem cells. HIF-2α downregulates P53 activity under hypoxic conditions and regulates cell proliferation, angiogenesis, metabolism, metastasis, and resistance to chemotherapy as a part of tumorigenesis^[186]^. Overexpression of HIF-2α enhances the expression of the endothelial kinase receptor, Tie2. Tie2 helps to develop the embryonic vasculature, which persists in adulthood, and it increases cytokine protein levels and mRNA in endothelial cells, promoting angiogenesis and tumor growth^[187–189]^. HIF-2α overexpression inhibits xenobiotic sensing nuclear receptors and their gene expression, affecting the expression of MDR1 and Cytochrome P450 3A4 (which oxidizes small foreign organic molecules expression). Furthermore, HIF-2α overexpression reduces the pharmacological effects of paclitaxel, mitomycin C, imatinib, and sorafenib on gastric cancer cells^[186]^. Currently, the evaluation of PT2385, a HIF-2α inhibitor, in combination with nivolumab targeted therapy to programmed death receptor-1 (PD-1) in patients with advanced clear cell renal cell carcinoma previously treated with one VEGFR targeted therapy is in clinical trial Phase I (NCT02293980). The combination of both drugs has demonstrated promising anti-tumor activity in ccRCC patients^[190]^. EZN-2208 is a transcriptional inhibitor of HIF-1α, which in combination with All-trans retinoic acid-arsenic trioxide (ATRA-ATO) was highly effective in treating patients with acute promyelocytic leukemia who develop resistance to ATO^[138]^.

In general, the downregulation of HIFs in tumors overexpressing this protein can be another strategy to prevent tumor drug resistance. A decrease in HIF-2α activity can be acheived by drug delivery strategies that introduce small molecule inhibitors of HIF-2α, interference RNA, or by inhibiting its downstream effectors.

### Breast cancer gene

There are two breast cancer genes, BRCA1 and BRCA2, and each one has different tumor suppressor characteristics. Their main function is to indirectly maintain the genomic integrity collaborating with recombination repair proteins^[191]^. Estrogen receptor signaling is the guardian of genome stability, together with the BRCA genes and proteins that control and repair DNA damage^[192]^. Both (BRCA1 and BRCA2) form complexes with Rad51, a recombination protein that controls the S/G2 phase in the cell cycle process. The BRCA proteins also form complexes with each other to collaborate in the tumor suppression process^[191]^. The BRCA1 performs several tasks, including DNA replication, cell cycle control, apoptosis, regulation of transcription, and chromatin unfolding^[193]^. Concurrently, BRCA2 activity is mainly focused on DNA repair by Rad51-mediated homologous recombination. When BRCA1 or BRCA2 genes are mutated, cancer cell lines diminish the DNA double-strand break repair ability through the process of homologous recombination (HR), promoting tumorigenesis due to genome instability^[191,194]^. When patients exhibit a BRCA mutation, they usually reveal p53 mutations as well. As previously discussed, p53 gene mutations prevent further p21 expression, favoring BRCA-mutated cells to avoid apoptosis, and perpetuate the development of cancer tumors^[195]^. Mutations in BRCA2 increase the risk of developing breast, prostate, pancreas, gall-bladder/bile duct, stomach and malignant melanoma^[191]^. Meanwhile, BRCA1 mutations increase the incidence of ovarian cancer and breast tumors^[196]^.

BRCA1 gene upregulation is caused by estrogen-induced cell proliferation and differentiation, supporting the effect of DNA stabilization. Upregulation of estrogen receptor expression is inhibited when BRCA genes are mutated, repressing the estrogen receptor’s function. Simultaneously, BRCA gene mutations upregulate defective estrogen signaling that leads to tumorigenesis^[192]^. CHD4 acts as a tumor suppressor gene in female cancers (i.e., ovarian cancer), promoting DNA repair similar to the BRCA gene, reducing proliferation, and increasing sensitivity to DNA damaging agents. CHD4 modulates therapeutic responses to DNA-damaging agents in BRCA mutant cancer cells. A previous study from Guillemette *et al.*^[197]^ (2015) revealed that mRNA expression levels from CHD4 contribute to the prediction of BRCA mutation cancers. When BRCA-associated cancer exhibited CHD4 depletion, a DNA-damaging agent (e.g., cisplatin) resistance was observed.

Meanwhile, the downregulation of p53 transcriptional activity is related to the overexpression of BRCA2^[198]^. In another study, BRCA2 inactivation decreased cell cycle progression and DNA replication and lowered cell proliferation compared to BRCA. BRCA2 knockdown is related to an innate immune response upregulation, promoting cell survival^[199]^. The anticancer drugs commonly used to treat breast cancer are taxanes and platinum agents. Taxane drugs include paclitaxel and docetaxel for BRCA1 gene mutations or hormone-negative cancers. The positive-hormone cancers are less sensitive to taxanes. Thus, platinum agent anticancer drugs such as cisplatin and doxorubicin are included as an alternative to triple-negative breast cancer (lack of estrogen receptors, progesterone receptors, and ERBB2 receptors)^[195]^.

### Occludin

Tight junctions (TJs) are structural proteins that control transportation across the cell membrane. These proteins regulate cellular permeability while maintaining cell polarity, restricting the diffusion of molecules through the membrane. Tight junctions also control cellular functions, including cellular responses to environmental stimuli, intracellular gene expression, cell differentiation, and proliferation. TJs are composed of membrane proteins that can interact with adjacent cells, functioning as a barrier ^[200,201]^. An integral component of TJs that provides structure and function is the protein occludin, encoded by the occludin (OCLN) gene^[202,203]^. Occludin oxidizes NADH^[204]^, which is essential for TJ morphology, stability, barrier function, and localization of the plasma membrane on endothelial cells^[200]^. Occludin contains a transmembrane domain with four membrane-spanning regions and other protein domains such as a C-terminus coiled-coil domain to interact with other proteins^[200,203]^. OCLN’s protein expression can influence the development of several cancer types, including ovarian cancer^[200]^, lung adenocarcinoma^[203,205]^, and breast cancer metastasis^[206]^. Zhang *et al.*^[200]^ (2018) reported that OCLN overexpression increased transepithelial resistance, which indicates stronger TJs, while downregulation of OCLN resulted in a decreased cell to cell adhesion phenotype (a common characteristic of tumors). Another study reported that OCLN overexpression stimulates malignant growth of lung cancer cells, thereby promoting proliferation and blocking apoptosis^[203]^. On the other side, eliminating the OCLN gene has been shown to promote tumorigenic factors and reduce susceptibility to apoptosis in squamous cell carcinoma^[201]^. OCLN expression increases on A549 lung cancer cells promote their resistance to cisplatin, doxorubicin, and gemcitabine. As an anticancer drug resistance mechanism, there is an increased expression of OCLN in the TJs of lung cancer cells. The overexpression of OCLN induces drug resistance by inhibiting the flux of doxorubicin, thus lowering drug concentration within the cell. OCLN may not be related to cancer drug resistance acquisition directly, but it limits the chemosensitivity of anticancer drugs to lung cancer cells^[205]^.

In the A549 lung cancer cell line, OCLN knockdown was not related directly to their resistance to anticancer drugs, yet it suppressed their chemosensitivity on a multicellular spheroid assay. OCLN overexpression on A549 cells decreased doxorubicin permeability due to their effect on signaling pathways, lowering the drug’s accumulation and cytotoxicity, leading to anticancer drug resistance. Interestingly, spheroid cancer cells with an increased OCLN expression developed cisplatin resistance, showing the importance of this gene in MDR^[205]^.

## DRUG DELIVERY SYSTEMS USING NANOPARTICLES TO IMPROVE THE EFFECTIVENESS OF CHEMOTHERAPEUTIC DRUGS IN RESISTANT TUMORS

Researchers have adopted several strategies to incorporate carriers to deliver a drug or a combination of drugs intracellularly. The development of nanoparticles has become an outstanding application of nanotechnology into medicine, where a nano-sized carrier efficiently delivers its payload of anticancer drug moieties. Using this type of therapy, researchers and clinicians take advantage of the irregular vasculature of the tumor to selectively deliver the drug and diminish the drug’s toxic side effects^[207,208]^. Another important advantage supporting the use of nanoparticles as a drug delivery system in cancer therapy is that it overcomes drug resistance by deactivating or avoiding various drug efflux pumps^[209,210]^. This could be accomplished by designing a selective (targeted) uptake of an endogenously endocytosed compound and promoting an intracellular accumulation of the drug, driven by the delivery system.

A remarkable characteristic of most of the nanoparticles for drug delivery systems includes a spherical shape and a large surface area-to-volume ratio. This property allows the nanocarriers to be absorbed through the cell’s membrane while carrying an anticancer agent. Also, most nanocarriers’ surface provide an alternative to add modifications, thus improving the nanoparticle’s targetability. Chemotherapeutic nanocarriers have two major categories for both active-targeted and passive-targeted delivery systems: 1) inorganic nanocarriers (metal core) and 2) organic nanocarriers (polymers, lipids, or liposomes)^[57]^. Currently, all the clinically approved nanocarriers are passive-targeted delivery systems ^[208]^. However, the clinical approval of these DDS for cancer therapy was not based on their effect against anticancer drug resistance, but instead on their potential to specifically target tumors based on their irregular vasculature. Based on this, we focus our next sections on studies of organic and inorganic nanocarriers, which showed significant results against resistance.

### Organic nanocarriers

Nanoparticles containing an organic core are biocompatible, solid, and often biodegradable. Organic nanocarriers are accessible for synthesis and viable for surface modifications. These characteristics increase the efficiency and biodistribution of the delivery system^[211]^. Based on our knowledge, currently, all the FDA-approved nanoparticle-based drugs are in the category of organic nanocarriers, i.e., protein-based polymers and liposomes; also, various nanoparticle-based drugs are in clinical trials^[208]^. The following section will discuss organic nanocarriers and their applications in cancer resistance, which is triggered by the genes discussed.

#### Polymers

In polymer nanoparticles, anticancer agents can be encapsulated through conjugation, or polymer attachments can be added to promote their release after a stimulus-response^[57]^. Risnayanti and his collaborators incorporated polylactic-co-glycolic acid (PLGA) and carboxylic acid-based particles to encapsulate both MDR1 and BCL2 siRNA^[79]^. Their design tackled drug efflux and cell death defense pathways. This dual MDR1 and BCL2 siRNA-loaded PLGA nanoparticle system was a viable strategy to overcome the chemoresistance on ovarian cancer cells (paclitaxel-resistant cell line SKOV3-TR and cisplatin-resistant cell line A278O-CP20) by enhancing cellular drug sensitivity^[85]^. Wang Z *et al.*^[212]^ (2017) developed PLGA nanoparticles to encapsulate the anticancer drug Disulfiram to protect it from degradation due to its short half-life in the bloodstream. The nanoparticles were combined with copper, to inhibit liver cancer stem cells. In addition, the nanoparticles were combined with 5-fluorouracil, thus resulting in a synergistic cytotoxicity and anti-metastasis effect on a mouse model of liver cancer. Another research group’s delivery system used PLGA as a water-soluble carrier. Chang *et al*.^[213]^ (2013) designed nanoparticles to encapsulate curcumin, a low water-soluble compound with anti-tumor, anti-metastasis, and anti-angiogenesis properties. The curcumin nanoparticles were used to treat cisplatin-resistant human oral cancer cells. As a result, curcumin nanoparticles induced apoptosis of the resistant cancer cells and showed low cytotoxicity to normal human oral epidermal cells. Moreover, these curcumin nanoparticles caused DNA fragmentation, upregulation of caspase-3/9, cytochrome c, and Apaf-1, while increasing ROS levels, which are known to induce apoptosis. In addition, Bcl-2 was downregulated, and the protein and mRNA expression levels of MDR1 were suppressed.

In other studies, Xiao *et al.*^[214]^ (2015) designed a double functionalized PLGA nanoparticle delivery system that included chitosan (to enhance endocytic uptake) and two drugs - Pluronic (an MDR1 inhibitor) and Camptothecin (a topoisomerase 1 inhibitor) - that were encapsulated into the nanoparticle to treat colon cancer, both *in vitro* and *in vivo.* The outcomes for this nanocarrier design were advantageous due to the downregulation of MDR1 expression and the inhibition of P-gp, which induces apoptosis and reduces systemic toxicity. Another study registered hyaluronic acid-modified Paclitaxel nanoparticles to encapsulate and deliver MDR1 siRNA inside ovarian cancer cells. This formulation inhibited tumor growth and induced apoptosis by decreasing P-gp and MDR1 expression. These particular nanoparticles take advantage of the cluster of differentiation 44 (CD44), targeting the hyaluronic acid receptor^[121]^. This active targeting strategy is an additional feature to increase drug accumulation into cells, promoting nanoparticle endocytosis instead of drug internalization by influx pumps. Finally, synthetic and natural polymers can be used to encapsulate and deliver drugs to overcome MDR.

#### Liposomes

Liposomes are drug delivery nanocarriers that exhibit biodegradable and biocompatible properties, with the ability to encapsulate water-soluble agents, e.g., DNA and RNA, in their aqueous inner core and insoluble agents into their bilayer membrane^[57]^. Those specific characteristics make the liposome a versatile therapeutic nanocarrier. In this way, a research group worked on a self-assembling nanocomplex to carry the p53 gene and targeted glioblastoma multiforme. They reported a cationic liposome composed of 1,2-dioleoyl-3-trimethylammonium propane (DOTAP) and di-oleoyl phosphatidylethanolamine (DOPE) as the carrier encapsulating an oligonucleotide. This nano-delivery platform successfully crosses the blood-brain barrier to target glioblastoma multiforme (GBM) and cancer stem cell lines: U87, T98G, and LN-18. The nanoparticles carried p53 and Temozolomide. This treatment revealed an increase in anti-tumor efficiency, increased sensitivity of cancer stem cells and tumor cells to the drug, activation of apoptosis, and decrease in cancer drug resistance in human cancer^[215]^. In another recent study, researchers developed curcumin loaded into a cationic liposome-polyethylene-glycol-polyethyleneimine complex (LPPC), together with the drug Herceptin (Trastuzumab) non-covalently intercalated on the surface of the carrier. Curcumin-LPPC-Herceptin and doxorubicin-LPPC-Herceptin complexes dramatically enhanced the cytotoxic effects of LPPC-encapsulated curcumin on HER2-positive cells, with a potent therapeutic effect on SKBR3 (HER2 positive) as compared to Hs578T (HER2 negative) breast cancer cells^[216]^.

#### Micelles

Micelles as spherical drug nanocarriers are self-assembly systems of water-soluble components in an aqueous solution that results in a hydrophobic core and a hydrophilic shell. The hydrophilic shell can stabilize the hydrophobic core while keeping a non-water-soluble drug inside. The resulting nanoparticle is an excellent candidate to carry non-water-soluble drugs that can be incorporated in the polymeric micelle through physical, chemical, or electrostatic interactions^[211]^. In a study, researchers prepared micelles of mPEG modified with transferrin and containing R547 as a drug delivery system. R547 is an ATP-competitive CDK inhibitor that specifically induces cell-cycle arrest and apoptosis. However, R547 is poorly soluble in an aqueous solution at physiological pH conditions, making it a candidate molecule to be integrated into a drug delivery system. These transferrin-modified micelles showed cytotoxicity against ovarian carcinoma cells, A2780, and inhibited tumor growth on A2780 tumor-bearing mice compared to non-drug and non-modified micelles^[217]^. Another research group showed the synergistic effect of using the combination of verapamil (P-gp inhibitor) and paclitaxel targeted-delivery into breast carcinoma cell lines, using a folate-conjugated deoxycholic acid micelle to overcome MDR. The cells used were MCF-7 and MCF-7/ADR (multi-drug-resistant variants). Synergistic effects of the folate receptor, whichmediates internalization, and the drugs diminished MDR. Side effects and toxicities to healthy tissues or organs were reduced^[50]^. In another study, a preparation of Herceptin conjugated to micellar nanoparticles consisting of d-a-tocopherol polyethylene glycol succinate (TPGS) was evaluated for the concomitant targeted delivery of docetaxel drug and the Polo-like kinase 1 siRNA to MCF7 and SKBR3 breast cancer cells. The synergistic effects of the co-delivery of drugs into the cells with different HER2 expression levels resulted in a sustained and controlled delivery of docetaxel, thereby increasing its therapeutic effect^[218]^. Finally, theranostic iron oxide-coated nanoparticles combined with cisplatin and with a tumor imaging infrared-dye- labeled HER2 antibody were presented in an interesting study. The study used HER2-positive chemo-resistant ovarian cancer cells (SKOV3) in female athymic nude mice. The results of this *in vivo* study showed inhibition of primary tumor growth and metastasis, and the downregulation of HER2 in an ovarian cancer xenograft model^[219]^.

#### Solid lipid nanoparticles

Solid lipid nanoparticles (SLN) are nanocarriers parallel to liposomes and lipid emulsions. SLN can incorporate drugs and perform targeted and controlled drug delivery^[220]^. Eskiler *et al.*^[221]^ (2018) developed solid lipid nanoparticles to treat resistant triple-negative breast cancer that is due to BRCA1 mutation. These nanoparticles are composed of Poly (ADP-ribose) polymerase (PARP) inhibitors to induce DNA damage and overcome HR-mediated resistance in HCC1937 and HCC1937R cell lines while delivering the anticancer drug Talazoparib (BMN673). PARP is a family of proteins associated with the regulation of many cellular processes such as genomic stability, DNA repair, and apoptosis^[222]^. The results from this study indicated DNA double-stranded breakage, G2/M cell cycle arrest, and PARP cleavage^[221]^. Differently, Choi *et al.*^[223]^ (2008) reported a cationic SLN design to deliver a non-viral vector-mediated p53 gene into H1299 lung cancer. SLN enters the cell to deliver the p53 gene through cell membrane permeabilization. After treatment, the *in vitro* and *in vivo* results showed an increase and restoration of p53 function and apoptosis, and decreased cancer cell growth. These nanoparticles reported higher efficiency of p53 gene delivery than wild type p53 mRNA and protein expression levels in lung cancer cells. The nanoparticles also promoted lung cancer cell restoration of apoptotic pathways and reversed deficiencies in both *in vitro* and *in vivo* tumor models^[223]^.

### Inorganic nanocarriers

Inorganic nanocarriers have shown various advantages over organic nanocarriers, including, for example, high stability on most organic solvents, large surface area, superior drug loading capacity, enhanced bioavailability, low toxicity, and controlled drug release^[57]^. Based on our knowledge, to date, there are several inorganic DDS under clinical trials for cancer therapy, i.e., gold nanoparticles, but none of them have been approved yet^[208]^.

A study incorporated an inorganic material carrier (i.e., nanodiamond) to effectively deliver the anticancer drug Epirubicin to the hepatic cancer stem cell line LT2-MYC (murine hepatoblastoma)^[224]^. These nanodiamonds reduced toxicity primarily through passive targeting so as to increase tumor-specific drug accumulation. This nanodiamond-Epirubicin complex exhibited high stability and adsorption, and promoted a significant uptake and retention of the drug in tumor cells. Also, these nanodiamonds prevented the efflux of Epirubicin by ABC transporters, enhancing drug retention that led to overcoming resistance triggered by the CHD4 gene^[224]^. In contrast, Zhang *et al.*^[225]^ (2020) developed PEGylated tetrasulfide organosilica shell nanoparticles, exploring the co-delivery of cisplatin and Acriflavine drugs to suppress HIF functions and inhibit metastasis. This delivery system was able to synergistically co-deliver the drugs into A549 adenocarcinoma lung cancer cells *in vitro* and *in vivo.* The results revealed the versatility of this system to combat anticancer drug resistance^[225,226]^. These nanocarrier designs demonstrate the effective incorporation of inorganic materials as a viable method to overcome anticancer drug resistance.

#### Transitional metals

Another type of inorganic nanocarriers include transitional metals. Most nanomaterials are metallic compounds; because of their inherent properties as nano-sized particles, they facilitate transportation through biologicals barriers. In this regard, nickel (Ni) is considered as a highly abundant metallic material candidate to be developed for health-based applications. Ingestion, inhalation, and skin absorption are the best routes for nickel to enter the human body, making the lung and kidney its primary targets^[227]^. Researchers have studied Ni-containing nanoparticles. Bioavailability and toxicological properties of metallic Ni nanoparticles and NiO nanoparticles have been examined on lung cancer cells. NiO nanoparticles promoted nuclear translocation of HIF-1α, consequently leading to the upregulation of NDRG1 in H460 human lung epithelial cells. However, these metallic nanoparticles showed moderate toxicity. Both Ni nanoparticles generated the activation of apoptosis through caspase and poly (ADP-ribose) polymerase^[228]^. On the other hand, a study using zinc oxide nanoparticles as treatment reported the upregulation of NDRG1 expression, and other cell growth and differentiation proteins essential in specific pathways to the ovarian granulosa cells of hens^[229]^.

Some studies have been incorporating metallic-based nanomedicine to treat cancer resistance caused as a response to hypoxia. Silver nanoparticles were incubated with the MCF7 (breast cancer cells) and HeLa (ovarian cancer cells) in hypoxic conditions. These silver nanoparticles promoted the inhibition of HIF-1 reporter and vascular endothelial growth factor (VEGF) to disrupt angiogenesis. In addition, silver nanoparticles disrupted the cellular function of HIF signaling pathway^[230]^. Satapathy *et al.*^[231]^ (2013) reported the implementation of silver-based nanoparticles as an alternative to treat HCT116 human colon cancer cells. These nanoparticles contributed to growth inhibition and increased cytotoxicity. This research group reported an increase in BAX/BCL-XL ratio, and p53, p21, caspase 3, 8, and 9 activations, leading to apoptosis; as well as a decrease in AKT and NF-κB levels^[231]^. The NF-κB and AKT levels were determined due to their importance in cell proliferation, promoting survival in resistant cancer. Silver-based nanoparticles can also be considered as an anticancer strategy to treat p53-dependent cancer cell development.

In addition, the study of titanium dioxide nanoparticles to determine their effects on the blood-brain barrier has increased. Disdier *et al.*^[232]^ (2015) study revealed that titanium (Ti) is internalized in the liver, lungs, and spleen, persisting for up to a year. However, in brain epithelial cells, Ti circulated for a short period and had no effect on blood-brain barrier integrity, although brain inflammation was reported. Interestingly, the presence of Ti in the liver increased TJ protein concentration, including OCLN, and promoted the modulation of P-gp mRNA expression^[232]^. Although the principal objective of this study was to analyze the effect of nanoparticles on the blood-brain barrier, their results showed the influence of Ti on the regulation of OCLN. The incorporation of Ti nanoparticles to influence cancer resistance by the modulation of OCLN protein has excellent potential for future studies.

In parallel to all the results presented in this section, the incorporation of nanomedicine to increase intracellular drug concentration, changing the cell uptake route of the drug (e.g., endocytosis instead of passive diffusion), and targeting cancer resistance-related genes show great potential as the next steps to improve cancer therapy. Due to this, there are many nano-sized DDS under different stages of translational research for cancer therapy.

Tumors are very heterogeneous in their cell population. In this way, the physiological barriers, e.g., microenvironment, that must overcome these DDS are more complex than challenges seen in the use of in-vitro models. In addition, it is still unknown whether the inherent physicochemical impact of the DDS nanoparticles inside the human body will completely change the fate and therapeutic effect of the drug. Thus, the uptake of drugs delivered by nanocarriers is not significantly higher than that of the free chemotherapeutic drug in patients. Due to this, researchers are still working on the development of more robust DDS to overcome these limitations.

Table 4 summarizes the different nanocarriers that have been studied to treat cancer resistance triggered by genes.

## CONCLUSION

Resistance to therapy continues to be the most significant medical challenge in cancer today. The current multimodal approach of cancer treatment is not enough to cure many tumor types and to decrease relapse. Since there are many underlying mechanisms of resistance, it is vital to understand the biological determinants. Identifying the biological drivers of drug resistance will result in new therapeutic strategies, which can focus on targeting the internal tumor characteristics that develop malignancies. The introduction of targeted and specific drug delivery systems such as nanocarriers is a big step towards the correct path in drug design. Non-toxic and targeted new cancer treatment alternatives will help overcome anticancer drug therapy resistance, thus providing hope to patients who are victims of this devastating disease.

## Figures and Tables

**Table 1.** Common classes or anticancer drugs

Classification	Mechanism of action	Type of drug	Examples of drugs	Cancer type	Ref.
Alkylating agents	Add alkyl groups to guanine on DNA; create cross links within the DNA	Platinum-based agents	Cisplatin Carboplatin Oxaliplatin	Breast, Leukemia, Lymphoma, Multiple Myeloma, Sarcoma, Brain Cancer, Ovary, Lung	[8–10]
		Nitrogen mustards	Chlorambucil Cyclophosphamide		
		Alkylsulfonates	Busulfan		
Antimetabolites	Interfere with vital metabolic pathways by acting as a false substrate during cell cycle synthesis phase	Pyrimidine antagonists	5-Fluorouracil Gemcitabine	Leukemia, Breast, Ovary, Intestinal Tract, Pancreatic, Colorectal	[11–14]
		Purine antagonists	Fludarabine		
		Purine analogs antifolates	6-Mercaptopurine Methotrexate		
		Ribonucleotide reductase inhibitors	Hydroxyurea		
Mitotic spindle inhibitors	Inhibit microtubule polymerization causing disruption of mitotic spindle formation	Taxanes	Paclitaxel Docetaxel	ALL, Burkitt lymphoma, Hodgkin lymphoma, Neuroblastoma, Rhabdomyosarcoma, Wilms tumor, NSCLC, Ovarian, Plead and neck	[14,15]
		Vinca alkaloids	Vincristine Vinblastine		
Topoisomerase inhibitors	Prevents resealing of DNA breaks	Topoisomerase inhibitors I and II	Topotecan Etoposide	Leukemia, Lung, ovarian, gastrointestinal, and other cancers	[14,16]
anti-tumor antibiotics	Different mechanisms (free radical formation, lipid peroxidation, direct membrane effects, and enzyme interactions)	Anthracyclines	Doxorubicin Daunorubicin	ALL, AML, Hodgkin’s and non-Hodgkin’s lymphoma, Bladder, Breast, Metastatic cancers, Esophageal	[14,17,18]
Tyrosine kinase inhibitors	Blocks the action of tyrosine kinases	Small molecules inhibitors	Erlotinib Lapatinib Ripretinib	Breast, CML,NSCLC, Lung, Renal, Hepatocellular, Prostate, Renal, Colorectal, ALL, GIST	[19–22]

Abbreviations: ALL: acute lymphocytic leukemia, AML: acute myeloid leukemia, CML: chronic myelogenous leukemia, NSCLC: non-small-cell lung carcinoma, GIST: gastrointestinal stromal tumors

**Table 2.** Common chemotherapeutic drugs associated with cancer cell resistance^#^

Chemotherapeutics	Cell cycle phase disrupted	Cellular pathways affected	Main resistance-related genes	Ref.
Cisplatin	DNA repair / Any phase of the cell cycle	-DNA damage DNA-platinum adducts leading to apoptosis	BRCA (1 and 2)- DNA damage repair	[30–33]
Doxorubicin	DNA replication and DNA repair	-DNA structure changes	BCL-2- Cardiotoxicity to non-cancer cells	[34–37]
		--Formation of free radicals and oxidative damage		
Paclitaxel	Mitosis	-Cytoplasmic microtubule-assembling disruptor	MDR1- Overexpression of P-gp, drug target alteration	[7,38,39]
		-Cell replication inhibitor		
5-Fluorouracil	DNA synthesis (DS)	-DS inhibition through thymidylate synthase targeting	BCL-2, Bcl-XL and p53 overexpression	[40–42]
		-Cell growth inhibition leading to apoptosis	-drug inactivation	
		-DNA and RNA damage	-drug target alteration	

# This is not an extensive list of all the drugs in chemotherapy that have acquired resistance. This table presents a list of the most well-known anticancer drugs used in chemotherapy for more than 20 years. BRCA: breast cancer genes; MDR1: multidrug resistance gene or P-glycoprotein-1; BCL-2: B-cell lymphoma 2

**Table 3.** Mechanisms of anticancer drug resistance

Mechanism	Short description	Ref.
Drug inactivation	Cancer cells generate an alternative mechanism that inactivates the drug that is inside the cell, contributing to modification, degradation, or complex formation. This inactivation decreases the drug’s toxicity levels, and reduces the damage and activity of the drug in cancer cells	[26]
Alteration of drug target	Altered or unrecognized protein structure in the drug’s transporter protein due to accumulated mutations can prevent proper attachment of the drug on its binding site. As a consequence, cancer cells become unable to internalize the cytotoxic drug, leading to their survival	[7,25]
Enhanced efflux pumps	The anticancer drug is pumped out of the cell through a transmembrane protein (efflux pump), preventing the accumulation of the effective drug concentration from causing toxicity in the cell, sabotaging the therapy	[26,29]
DNA-damage repair	Cancer cells may gain the ability to repair the DNA damage/breakage caused by anticancer drugs as a response to promote cell survival	[7,29]
Cell death inhibition	When proteins that induce cell death pathways (apoptosis, necrosis, or autophagy) are mutated or altered, they are unable to induce cell death	[52]
Tumor cell heterogeneity	Cancer cells multiply at an uncontrolled rate, accumulating genetic mutations and epigenetic changes, which lead to resistance and affect their sensitivity to cancer drugs. The generation of cell heterogeneity leads to the development of stem cell-like properties on the new growing cells. The stemness effect is common in cancer cells that are in circulation	[53]
Genetic factors	Include gene mutations, amplifications, and epigenetic alterations. Epigenetic events such as methylation and acetylation affect genetic expression leading to the silencing, overexpression, or amplification of oncogenes or tumor suppressor genes, resulting in the development of cancer drug resistance	[54]

**Table 4.** Drug delivery system nanoparticles^#^ and their effect on cancer resistance

DDS carrier	Nanoparticles modification	Encapsulated drug or toxic agent	Cells or tumor treated	Genes affected	Effect over resistance	Ref.
PLGA^*^	Dual RNAi delivery system (MDR1 and BCL2 siRNA)	Paclitaxel^*^ and cisplatin^*^	ovarian cancer cells: SKOV3-TR and A2780-CP20	MDR1 and BCL2	Stimuli inhibition of drug efflux and cell defense pathways (enhanced drug sensitivity)	[85]
PLGA^*^	PLGA-encapsulated Disulfiram	Disulfiram^*^	Hepatocellular carcinoma ( Huh7, PLC/PRF/5)	CHD4	Extended the half-life of Disulfiram	[212]
PLGA^*^	Pluronic and chitosan surface-functionalized PLGA nanoparticles	Camptothecin^*^	Colon-26 cells (Colon cancer cells)	MDR1	Downregulate the expression of MDR1 expression and enhanced tumor uptake. Induced tumor cell apoptosis, reduced systemic toxicity, and inhibited P-gp.	[214]
PLGA^*^	PLGA-curcumin nanoparticles	Curcumin^*^	CAL27-cisplatin-resistant human oral cancer cells	MDR1 Bcl-2	Suppress the protein and mRNA expression levels of MDR1.	[213]
					Downregulate the protein levels of Bcl-2. Intrinsic apoptotic pathway through regulating the function of MDR1 and the production of ROS	
PEG^*^ and PEI	hyaluronic acid (HA) based nanoparticle	MDR1 siRNA with paclitaxel^*^	SKOV-3TR and OVCAR8TR Ovarian cancer cells	MDR1	Down-regulation of MDR1 and P-gp expression. Inhibitory effect on the tumor growth. Decreased P-gp expression and increased apoptosis in MDR ovarian cancer mice model	[121]
ModifiePEG-PE micelles	Tf-conjugated polymeric micelles	R547 (a potent and selective ATP-competitive CDK inhibitor)	A2780 ovarian carcinoma cells	P21	*In vitro* and *in vivo* studies in ovarian cancer confirmed cytotoxicity and tumor growth inhibition.	[217]
Deoxycholic acid micelles	Folate-conjugated	Verapamil^*^, a P-gp inhibitor, and Paclitaxel^*^	MCF-7 and MCF-7/ADR (multi-drug-resistant variant), human breast carcinoma cell lines	MDR and P-gp	Verapamil-mediated overcome MDR solid tumors by targeting the delivery of micellar Paclitaxel into tumor cells.	[50]
Cationic liposome DOTA/DOPE^*^	systematic nanodelivery platform encapsulating human p53 or oligonucleotide	Temozolomide^*^ and p53 therapy	Human GBM cell lines U87, T98G, and LN-18	p53	DDS crosses the blood-brain barrier and efficiently targets cancer stem cells and tumor cells, activating apoptosis.	[215]
Cationic liposome-PEG-PEI complex	Herceptin was non-covalently associated onto the surface of the nanocarrier	Curcumin^*^ and doxorubicin^*^	SKBR3 ( HER2-positive) and Hs578T ( HER2-negative) breast cancer cells	HER2	Cytotoxicity improved. Anti-proliferative effect increased.	[216]
Micells TPGS^*^ and siRNA	Herceptin-conjugated micelles	Docetaxel^*^ and polo-like kinase 1 siRNA	MCF7 and SK-BR-3 cell lines Breast cancer cell	HER2	Co-delivery of drugs was sustained and controlled	[218]
amphiphilic polymer nanoparticle	coated magnetic iron oxide	Cisplatin^*^ and near-infrared dye labeled HER2 antibody	SKOV3 ovarian cancer cell line. *In vivo* models female athymic nude mice	HER2	Inhibited the growth of the primary tumor, peritoneal, and lung metastasis in ovarian cancer. Shrinkage of tumor and primary tumors that had low levels of HER2.	[219]
Nanodiamond	Epirubicin^*^ nanodiamond complex	Epirubicin^*^	LT2-MYC cell line from murine hepatoblastoma tumor model	CHD4	Nanodiamond-drug complex with epirubicin exhibited high stability and adsorption, promoting uptake and retention on tumor cells	[224]
Nickel oxide	Nickel-containing nanoparticles		H460 human large cell lung cancer	NDRG1 and HIF-1a	Activate a toxicity pathway characteristic of carcinogenic Ni compounds	[228]
Zinc oxide	ZnO nanoparticles		Jinghong-1 laying hen’s ovarian granulosa cells	NDRG1	Upregulated the expression of NDRG1 and regulate proteins	[229]
Silver nanoparticles	Ag nanoparticles		MCF7 (breast cancer) and HeLa (cervical cancer) cells	HIF-1	HIF-1a signaling pathway disrupted and vascular endothelial growth factor to inhibit angiogenesis.	[230]
Silica matrix	microporous organosilica shell-coated cisplatin nanoparticle^*^	Cisplatin^*^ and acriflavine	A549 lung cancer cells	HIF-1	Synergistic co-delivery of drugs. Inhibit metastasis and enhancing cisplatin efficiency	[226]
Solid lipid nanoparticles	PARP inhibitor to induce toxicity	Talazoparib^*^ (BMN 673)	HCC1937 and HCC 1937R Triple-negative breast cancer	BRCA1	DNA double-stranded breakage, G2/M cell cycle arrest and PARP (protein regulator of genomic stability) cleavage	[222]
Titanium dioxide nanoparticles	TiO_2_^*^ nanoparticles	Titanium	Brain epithelial cells (brain microvasculature endothelial cells) and Male Fisher F344 rats	OCLN	Occludin protein is regulated while crossing blood-brain barrier with not affected integrity. Upregulation of tight junction proteins, modulation of P-gp mRNA expression	[232]

* Food and Drug Administration (FDA) approved drug, polymer of particle.

# All these DDS have been tested *in vitro, in vivo* or both, but none of them have been FDA approved. Abbreviations: PLGA: poly(lactic-co-glycolic acid); RNAi: RNA interference; siRNA: small interfering RNA; PEG: poly(ethylene glycol); PEI: polyetherimide; DOTA/DOPE: 1,2-dioleoyl-3-trimethylammonium propane/di-oleoyl phosphatidyl ethanolamine; TPGS: d-α-tocopherol polyethylene glycol succinate; PE: phosphatidylethanolamine; PARP: poly ADP ribose polymerase; BRCA: breast cancer gene; MDR1: multidrug resistance gene or P-glycoprotein-1; HIF: hypoxia-inducible factor; OCLN: occludin
